# Assessing the efficacy of acupuncture as the sole analgesic for canine chronic pain

**DOI:** 10.18849/ve.v9i1.681

**Published:** 2024-03-06

**Authors:** Jianjian Gong+, Mary Frances Thompson+

**Keywords:** ACUPUNCTURE, ALTERNATIVE MEDICINE, ANALGESIA, CANINE, CHRONIC PAIN, DOG, PAIN MANAGEMENT

## Abstract

**PICO question:**

In dogs with chronic pain, is acupuncture alone, compared to a placebo, more efficacious in alleviating pain and pain-related dysfunction?

**Clinical bottom line:**

**Category of research:**

Treatment.

**Number and type of study designs reviewed:**

Four randomised, placebo-controlled clinical trials were critically appraised.

**Strength of evidence:**

Weak.

**Outcomes reported:**

A single study evaluating the efficacy of gold bead implantation, a form of permanent acupuncture, on pain associated with canine hip dysplasia (CHD) endorsed acupuncture’s superior pain alleviation and locomotion improvement through owner and veterinarian subjective outcome evaluation.

Three studies concluded that, overall, acupuncture was not efficacious regarding pain reduction or dysfunction improvement compared with placebo treatment.

**Conclusion:**

Based on the limited current evidence, acupuncture could have analgesic effects as perceived by owners, but acupuncture, as a sole analgesic, is unlikely to be effective in alleviating pain and pain-related dysfunction in canine chronic pain associated with musculoskeletal causes. Evidence is lacking on chronic pain due to neurological and oncological causes. Further studies need to focus on researching various acupuncture modalities’ effects on chronic pain with musculoskeletal, neuropathic and oncological causes when utilised as a component of multimodal therapy. Currently, for canine patients with chronic pain, there is insufficient evidence for a veterinarian to recommend that a client utilise acupuncture as the sole method for pain management.

## How to apply this evidence in practice

The application of evidence into practice should take into account multiple factors, not limited to: individual clinical expertise, patient’s circumstances and owners’ values, country, location or clinic where you work, the individual case in front of you, the availability of therapies and resources.

Knowledge Summaries are a resource to help reinforce or inform decision-making. They do not override the responsibility or judgement of the practitioner to do what is best for the animal in their care.

## Clinical scenario

You are a small animal primary care veterinarian and a 2-year-old neutered male Labrador Retriever presents with chronic pain associated with hip dysplasia of 5 months’ duration. The dog’s owner requests a conservative management plan. Two nonsteroidal anti-inflammatory medications (NSAIDs) have been trialled, but neither was effective in controlling the pain, and the dog suffered significant diarrhoea presumed to be a side effect of the medications. The owner is reluctant to pursue further treatment with medications and would like to trial acupuncture. Should you, as a veterinarian, endorse acupuncture as the sole analgesic for this dog?

## The evidence

Four randomised, placebo-controlled clinical trials that addressed the PICO question were identified, of which three investigated the efficacy of gold wire / bead implantation and dry needle acupuncture on pain associated with canine hip dysplasia (CHD) (Hielm-Björkman et al., 2001; Jæger et al., 2006; and Teixeira et al., 2016) and one explored the efficacy of electroacupuncture on pain associated with elbow joint arthritis (Kapatkin et al., 2006).

## Summary of the evidence

**Hielm-Björkman et al. (2001):** 

Population	Recruitment: Client-owned dogs selected using a questionnaire mailed to owners regarding the history, canine hip dysplasia (CHD) diagnosis, previous treatment, pain, and behaviour. Inclusion criteria: Unilateral / bilateral CHD. At least two of the following signs: difficulties in lying down / rising from recumbency, difficulty in jumping / refusing to jump, difficulties in walking up and down stairs, unilateral / bilateral hindlimb lameness and / or signs of pain when stretching a hindlimb backwards. Exclusion criteria: Insufficient clinical signs, lameness due to pathology in a non-hip joint, hindlimb trauma, septic articular infection, lumbar disease, neurological deficits, systemic / infectious disease. Other population information: 24 breeds female (n = 17); male (n = 21) 1–11 years of age (average age is 5.1 years) 14–59 kg (mean = 31.1 kg) 32/38 dogs pre-study treatment with NSAIDs, corticosteroids, pentosan polysulfate sodium, acupuncture needling or surgery, and none of them had pain treatment for minimum 30 days prior to study commencement previous femoral head ostectomy (n = 3) pectineus myotomy at least 6 months before the study (n = 4) >2 years’ duration of pain (n = 15).
Sample Size	38 dogs.
Intervention Details	Dogs were randomly assigned to acupuncture (gold wire implantation) and placebo groups (19 dogs per group). The treatment was revealed to neither the veterinarians who checked the dog nor the dogs’ owners until the end of the study. Evaluated at 0, 4, 12, and 24 weeks by veterinarians and 0, 1, 2, 3, 4, 12 and 24 weeks by owners. Initial assessment: Physical, orthopaedic (hip extension test, awake hip subluxation test and sedated Ortolani test) and neurological (proprioceptive positioning, hindlimb withdrawal reflex and patella reflex) examinations. Evaluation of video recordings of dogs’ movements by two independent veterinarians. Ventral dorsal radiographs of hip joints at week 0 (diagnosis confirmation) and week 24. Acupuncture group: Ohmmeter-detected implant sites on a 15 cm × 15 cm surgically prepared area over both hip joints; patient in lateral recumbency. Acupuncture points: GB29, GB30 and BL54 for most dogs; 1–3 extra points were treated in four hip joints. 24k gold wire piece (length = 2 mm, diameter = 1 mm) implantation by certified veterinarians with 14-gauge hypodermic needles into the acupuncture points; held in tissue by sterilised stilettos. Wound cleaning with an antibiotic cream (Terra Poly Vet; Pfizer) and covering with elastic tape. Prophylactic antibiotic: 30 mg/kg procaine benzylpenicillin injection (Duplocillin LA vet injection; Gisbrocades) in the neck musculature. Placebo group: Three needle punctures with identical-sized needles at non-acupuncture points. Hindlimb movement examination before discharge. Rescue analgesia: Meloxicam (Metacam; Boehringer Ingelheim) at 0.1–0.2 mg/kg/day when owners considered necessary. Required usage documentation.
Study design	Randomised, controlled, double-blind clinical trial.
Outcome studied	Subjective veterinarian assessment: Locomotion: based on five scores evaluating walking, trotting, galloping, jumping on / off a table and climbing stairs; ‘improved’ if any of the five scores was improved and other scores did not deteriorate; ‘deteriorated’ if all scores deteriorated / some deteriorated and some unchanged; ‘no definitive change’ if no change/improvement and deterioration happened concurrently. Hip function: graded by observed pain and the range of motion when bending hindlimbs vertically backwards. Radiographic evaluation of osteoarthritic changes by a veterinarian from the Finnish Kennel Union. Subjective owner assessment: A questionnaire on dog’s status, locomotion and behaviour related to pain from the hip joint. Two visual analogue scales (VAS) scores for pain and quality of life, respectively with a scale from 0–10 (0 = no pain / no locomotion difficulty, 10 = worst pain / worst degree of locomotion), evaluated at weeks 0, 1, 2, 3, 4, 12 and 24. A question regarding dogs’ performances and locomotion status change, climbing stairs, and signs of pain at weeks 12 and 24.
Main findings (relevant to PICO question)	Two dogs in the placebo group were euthanised and removed between week 12 and 24 due to intense pain. No difference in locomotion improvement in veterinarian or owner assessment between the treatment groups (P = 0.19). No difference in locomotion improvement or deterioration by percentage of patients between treatment groups. No difference in owners’ VAS evaluation between treatment groups (P = 0.895). No differences in owner evaluation of dogs’ performances and status concerning locomotion, climbing stairs and signs of pain between treatment groups after 12 or 24 weeks (excluding results from previously surgical-treated dogs).
Limitations	Dogs previously treated with acupuncture not excluded. Underpowered study: number of dogs (n = 19) in each group was sufficient to enable detection of a 25% efficacy difference between gold wire implantation and placebo groups with a statistical power of 80% (α-error 5%). The resultant efficacy difference between gold wire implantation and placebo groups approximated 12%, and 60 dogs would be required per group to detect this modest effect size with the same power and error. One dog in each treatment group had pain for less than 1 month before entering the study. Number of dogs administered rescue analgesia in each group was not reported. Two dogs did not complete the study, which further reduced the sample size and influenced the power of the study. Sham acupuncture might not be a suitable placebo as it may have potential effects in ameliorating pain. Lack of objective pain measurements: subjective owner and veterinarian assessments could have been impacted by caregiver placebo effect. Veterinarian hip function evaluation solely through video recording, which could have caused inaccuracy. Improvement and deterioration of locomotion determined using a subjective threshold. Needles with larger diameters (1.2 mm) than regular acupuncture needles (0.25–38 mm) used. Repeated clinic visits could have meant that owners adopted more broad clinical suggestions (e.g., weight loss and exercise) to help dogs in both groups to improve and mask the treatment effects.

**Jæger et al. (2006):** 

Population	Recruitment: Family-owned dogs from Norway recruited through veterinary and breeders’ club magazines. Inclusion criteria: 1–8 years of age history of pain and / or lameness / hindlimb dysfunction due to canine hip dysplasia (CHD) no previous acupuncture treatment. Exclusion criteria: Dogs with other neurological, muscular, or skeletal system-related diseases. Other population information: Pre-study nonsteroidal anti-inflammatory medications (NSAIDs) and corticosteroid medication withdrawal for minimum 14 days and 3 months, respectively 28 breeds in total: 19 German Shepherds, 7 Golden Retrievers, 7 Labrador Retrievers, 6 mixed breed dogs and 41 dogs of other breeds Male (n = 33); female (n = 47) Mean bodyweight = 34.8 kg Average duration of apparent pain 2–3 years Norwegian Kennel Club-engaged veterinarian / a surgeon (for mixed breed dogs) evaluated the hips status for all dogs.
Sample Size	80 dogs.
Intervention Details	Treatments were blinded for both owners and veterinarians. Owners instructed to provide dogs with the same exercise and food and document any pre-study analgesics. Dogs allocated into two groups by block randomisation (four dogs / block) according to bodyweight and CHD severity (acupuncture group: n = 38; placebo group: n = 42). The assessment of each dog was done by the same investigator. Rescue NSAIDs were permitted when owners considered necessary; usage documentation was required. Dogs were evaluated before gold beads implementation, and at 2 weeks, 3 months, and 6 months. Acupuncture (gold bead implantation) group: Two gold beads (cut from a 24k gold wire, length = 2 mm, diameter = 1 mm, weight = 35–45 mg) packed and autoclave-sterilised with a stiletto, then implanted at five defined acupuncture points under fluoroscopy. Placebo group: Skin prepared at five non-acupuncture points without gold bead insertion.
Study design	Randomised, double-blinded clinical trial with stratification.
Outcome studied	Six-point Likert scale documenting overall response according to owners’ impression of changes in mobility, lameness, stiffness, and behaviours at home and during exercises. Subjective owner assessments: Two visual analogue scales (VAS) (pain and dysfunction). Quality of life based on overall wellbeing. Subjective veterinarian assessments: Hip score according to pain response when manipulating each hip joint. Total hip score by addition of hip scores for each leg. Total lameness score by addition of gradings of 5 gaits / dog: lameness evaluation through video recorded walking, trotting, before and after stretch / extension of each hip at each visit.
Main findings (relevant to PICO question)	Two dogs did not complete the study for non-treatment related reasons, and the results were based on 78 dogs. Greater percentage of dogs with improvement in acupuncture group (30/36 [83.3%], 95% confidence interval [CI] 67.2–93.6) after 6 months compared to placebo group (25/36 [59.5%], 95% CI 43.3–74.4) (P = 0.02); no difference between treatment groups after 14 days and 3 months. Higher initial main pain score in acupuncture group (5.6, 95% CI 5.1–6.0) than placebo group (4.8, 95% CI 4.5–5.2) (P = 0.02); no difference between acupuncture (2.6, 95% CI 1.8–3.3) and placebo group (3.1, 95% CI 2.3–3.8) after 3 months; lower main pain score in acupuncture group (1.9, 95% CI 1.3–2.5) after 6 months (P < 0.01). Larger pain score reduction percentage in acupuncture group (65.4%) than placebo group (35.9%) after 6 months (P < 0.01). Longer median period from treatment to pain reduction in acupuncture group (21 days, 95% CI 5–30 days) than placebo group (10 days, 95% CI 5–135 days) (P = 0.04). No initial mean dysfunction degree difference between the treatment groups; lower degree in placebo group (3.4, 95% CI 2.8–3.9) than acupuncture group (4.4, 95% CI 3.6–5.2) after 14 days (P = 0.01); lower degree in acupuncture group (1.6, 95% CI 1.0–2.1) than placebo group (2.4, 95% CI 1.7–3.2) after 6 months (P = 0.03). Higher dysfunction score reduction percentage in acupuncture group (64.6%, 95% CI 52.9–76.3%) than placebo group (39.3%, 95% CI 21.0–57.6%) after 6 months (P = 0.03). No quality of life difference between treatment groups. Larger reduction in veterinarian-assessed pain in acupuncture group after 6 months (P = 0.03).
Limitations	Underpowered study: number of dogs in the acupuncture group (n = 36) completing the study was less than pre-study power calculation of 40 dogs per group (power = 90%, improvement in gold implantation group = 60%; improvement in placebo group = 30% and P < 0.05). Recruitment method might have attracted more pro-acupuncture dog owners than impartial dog owners. Owner compliance regarding instruction to provide dogs with identical food and exercise after enrolment could not be controlled. Lack of objective pain and lameness evaluation. Dogs in acupuncture group had more severe initial pain and greater rest needs. Locations of the five non-acupuncture points used for sham acupuncture were not described. Subjective assessment of owners could have been impacted by caregiver placebo effect. The challenge of lameness evaluation through video recordings could have affected lameness grading accuracy of veterinarians. NSAID medication usage could have confounded the study outcome: NSAIDs were administered to four dogs in the acupuncture group and one dog in the placebo group during the first 4 weeks following treatment and eight dogs in the placebo group during the 3–6 months following treatment. The drop-out of two dogs further reduced the sample size, which could impact the power of the study. Amount of rescue NSAIDs given by owners could not be controlled. Multiple confounding factors (diet, exercise, nursing care etc.) could have impacted the study outcome considering the long follow-up period.

**Kapatkin et al. (2006):** 

Population	Recruitment: Client-owned dogs. Inclusion criteria: Elbow osteoarthritis (OA) evidenced by forelimb lameness and radiography. > 1 year duration of clinical signs. Other population information: Six Labrador Retrievers, one Golden Retriever, one German Shepherd and one mixed-breed dog Body condition: ≥ 5 (n = 3); < 5 (n = 6) Bilateral elbow (n = 8) and unilateral elbow (n = 1) OA Five dogs underwent surgical treatment when ≤ 1 year of age with owner-reported post-surgery improvement Three dogs had radiographic evidence of hip joint OA but no clinical evidence of hindlimb lameness One dog underwent surgery for bilateral cranial cruciate ligament tears > 1 year before study.
Sample Size	10 dogs.
Intervention Details	The population information related to the dog that did not complete the study was not reported. Dogs not given analgesic medications or nutraceuticals during the study. Constant dog activity maintenance and over-exercise avoidance requested of owners. Three-phase study with two groups: First phase: 2–3 weeks force plate acclimation without treatment; dogs trotted over a commercial force plate a minimum of three times at weekly intervals for force plate gait analysis through ground reaction force (GRF); recorded each limb’s mean GRF from the first five valid trials (valid trial = dog’s velocity at 1.6–9 m/s^2^; acceleration = ± 0.5 m/s^2^; and ipsilateral forelimb and hindlimb striking the force platform completely, confirmed by two investigators). Second and third phases: 3 week acupuncture treatment followed by 3 week sham treatment (n = 5); or vice versa (n = 4); force plate gait analysis before and between 1–3 hours after acupuncture /placebo treatment. Owners blinded to treatments in the second and third phases; acupuncturist blinded to gait analysis results; all force plate analyses performed by same investigator, blinded to treatments. Acupuncture (electroacupuncture) treatment: Electrical-stimulated acupuncture once a week (20 minutes) for 3 weeks. Acupuncture needles (0.22 x 25 mm) inserted at the THL3, THI0, LU5, PC3, PC6, HT7, Baihui, GB33, GB34, BL10, and GV14. Stimulation frequency = 2 Hz increased until local muscle contraction apparent, then maintained. Bilateral treatment for bilateral OA dogs. Placebo treatment: Sham acupuncture once a week for 3 weeks. Single, unstimulated dermal needle placed at top of dog’s head, away from acupuncture points recognised by the International Veterinary Acupuncture Society.
Study design	Randomised, controlled, double-blinded, single-crossover clinical trial.
Outcome studied	Objective force plate gait analysis: peak vertical force (PVF), vertical impulse (VI), peak braking force, braking impulse, peak propulsion force, and propulsion impulse. Subjective owner evaluation: one visual analogue scale (VAS) was used to evaluate each of the four parameters (pain severity at walk, after recumbency, getting up and own, and the dogs' general well-being), respectively, and comments were made regarding dog’s activity level during the previous week.
Main findings (relevant to PICO question)	One dog did not complete the study due to illness unrelated to forelimb lameness. Neither acupuncture nor placebo affected PVF, VI, peak breaking force, peak propulsion force or propulsion impulse of any limb. No significant interaction found between treatment and visit numbers for any of the four VAS scores. The majority (8/9) owners identified when their dogs had undergone acupuncture treatment, greater than the proportion based on chance alone (P = 0.04).
Limitations	Lack of positive control to verify GRF measurement sensitivity in detecting gait alteration. Very small sample size. Power calculation not provided. Exclusion criteria not described. Unreported baseline characters (age, bodyweight, OA severity): different baseline characters could have impacted study outcome. Undescribed pre-study medication: effects of pre-study medication could have extended into study period as a confounder. No veterinarian pain measurement. One dog did not complete study: missing one data point for a small sample (nine dogs) could potentially have impacted linear model analysis. Sham acupuncture might be a less ideal placebo due to potential analgesic effects. Blinding methods not described in detail. Most included dogs had previous surgical treatments, the effect of which might have been enduring.

**Teixeira et al. (2016):** 

Population	Recruitment: Dogs attending Universidade Estadual Paulista’s veterinary hospital. Inclusion criteria: Unilateral / bilateral canine hip dysplasia (CHD) confirmed by radiographs. Pain as assessed by owners. Lameness as evidenced by at least two of the following: difficulty in lying down / standing up / going up and down stairs / reluctance to jump or jumping difficulty. Exclusion criteria: Administration of analgesic or chondroprotective drugs during the 4 weeks before the study and / or previous hip joint surgery.
Sample Size	34 dogs.
Intervention Details	Dogs were randomly allocated into acupuncture (n = 17), and placebo (n = 17) groups. A further 20 dogs were randomly allocated to a carprofen group, but related data were not considered for the purpose of this appraisal. Owners were absent from intervention sessions and thus blinded to the treatment arm of their dog. Presence and severity of CHD confirmed and classified by a radiologist according to Orthopaedic Foundation for Animals’ scheme. Evaluation at weeks -2, 0 (baseline), 2, 4, 6. Acupuncture group: Dry needle acupuncture by two veterinary acupuncturists: 25 × 30 mm needles for five sessions in 30 days, no owner presence. Acupuncture points: KI 3, BL 11, 18, 23, 40, 54, LIV 3, GB 29, 30, 34, and a single point of GV 2 and Baihui Lactose capsule (1 mg/kg) PO daily. Placebo group: Attendance at acupuncture clinic once a week for five sessions within 30 days. No needle insertion. Lactose capsule (1 mg/kg) PO daily. Rescue analgesia: Tramadol hydrochloride 4 mg/kg every 8 hours when owners noted pain deterioration. Usage report required. Results included in analysis. Lameness evaluation (pressure platform): Five valid walks per dog at 0.9 to 1.1 m/s (valid walk = all limbs touched the platform minimum twice, no sudden head movement or pulling during walk).
Study design	Randomised, controlled, single-blinded clinical trial.
Outcome studied	Subjective owner evaluation: Canine Brief Pain Inventory (CBPI) (significant improvement defined as 30% decrease in total), Helsinki Chronic Pain Index (HCPI) and visual analogue scale (VAS) (pain and locomotion). Objective lameness evaluation: Ground reaction force (GRF) (hindlimb peak vertical force [PVF] and vertical impulse [VI] symmetry indices).
Main findings (relevant to PICO question)	Age (only dogs that completed the study were reported): Acupuncture group: 7.2 ± 3.1 years. Placebo group: 6.6 ± 4.7 years. Breeds (only dogs that completed the study were reported): Acupuncture group: one Boxer, three German Shepherds, three Labrador Retrievers, two Rottweilers and five mixed-breed dogs. Placebo group: one Belgian Shepherd, six German Shepherds, one Golden Retriever, five mixed breed dogs, one Poodle, and two Rottweilers. Bodyweight (only dogs that completed the study were reported): Acupuncture group: 34.9 ± 8.2 kg. Placebo group: 30.7 ± 8.3 kg. Sex (only dogs completed the study were reported): Acupuncture group: female (n = 10); male (n = 5). Placebo group: female (n = 9); male (n = 7). Removal of one dog from the acupuncture group due to development of neurologic signs. Removal of one dog from each of the acupuncture and the placebo group due to non-approved medication administration by owner and contact loss, respectively. No difference in HCPI, CBPI and VAS between acupuncture and placebo groups at any time point. Greater CBPI improvement in acupuncture group (13/15 dogs) than placebo group (7/16 dogs) (P = 0.034). No difference in PVF or VI between acupuncture and placebo groups.
Limitations	Inclusion of dogs treated with acupuncture before the study: lasting acupuncture effects and owners’ attitudes towards acupuncture could have influenced the subjective assessment. Pre-study average pain duration experienced by dogs not described. Large variations in dogs’ age, bodyweight, and body conformation within treatment groups. Uncontrollable owner compliance with instructions and rescue analgesic use as dogs taken home after treatment. GRF measurement sensitivity insufficient for detecting gait alteration between dogs with and without CHD. Conformation variation could have impacted VI measurement. The drop-out of dogs in both acupuncture and placebo groups reduced sample size and impacted power of the study. Underpowered study: number of dogs (n = 15) in acupuncture group completing study was less than pre-study power calculation (n = 16, power = 80% for standard deviation [SD] = 40% and P < 0.05, expected minimum differences of variables between groups = 30%). Rationale for designating 30% CBPI decrease from baseline as significant improvement was unexplained. Variations of CBPI, HCPI and VAS scores within each group not reported. Some dogs were not able to maintain trotting for gait analysis due to severe CHD. Measuring walking instead of trotting could have affected gait analysis accuracy.

## Appraisal, application and reflection

A universally accepted definition of ‘acupuncture’ has not been adopted. Which modalities are considered within the scope of ‘acupuncture’ varies amongst professional bodies and practitioners. In broad terms, ‘acupuncture’ is the stimulation of specific points in the body with the aim of achieving homeostatic or therapeutic effects. In veterinary medicine, various related modalities have been utilised, including dry needle acupuncture, electroacupuncture, aqua acupuncture, laser acupuncture and material implantation (Cantwell, 2010; and Roynard et al., 2018).

Four randomised, controlled clinical trials addressing the PICO question were identified, with the low number of trials constituting a very limited body of evidence. Treatment effects of three acupuncture modalities (dry needle acupuncture, electroacupuncture, and gold wire / bead implantation) in comparison to placebo on pain and pain-related dysfunction associated with two musculoskeletal conditions (chronic elbow joint arthritis and hip dysplasia) were examined. No placebo-controlled clinical trial addressing acupuncture’s efficacy as the sole analgesic on chronic pain associated with neurological or oncological causes was identified. Typical study participants were adult (> 1-year-old), medium- to large-sized dogs of varying breeds. All dogs were family-owned, thus home environment, diet and interaction with owners represented potential confounding factors, however, corresponds with authentic clinical practice. Three of the four studies were underpowered(Hielm-Björkman et al., 2001; Jæger et al., 2006; and Teixeira et al., 2016). In Jæger et al. (2006) and Teixeira et al. (2016), there were fewer dogs in the acupuncture treatment group than the minimal sample size according to the pre-study power calculation and in Hielm-Björkman et al. (2001), there were fewer dogs in the placebo group than the minimum sample required. A power calculation was not described in Kapatkin et al. (2006).

Jæger et al. (2006) concluded that gold bead implantation provided greater analgesic effects and improved mobility compared to placebo for dogs with hip dysplasia, and suggested the gold bead implantation should be considered when conservative / medical treatment is ineffective, or surgical intervention is not an option. It is the only study examined that was able to demonstrate superior analgesic efficacy of acupuncture. The findings contradict the similar study utilising gold wire implantation and placebo treatment (Hielm-Björkman et al., 2001), in which no difference in pain and dysfunction reduction was identified between group. Jæger et al. (2006) included a larger sample population (78 dogs) than Hielm-Björkman et al. (2001) (38 dogs), which could have assisted in identifying a treatment effect. Dogs in the former study were limited to 1–8 years of age, while the oldest dog in Hielm-Björkman et al. (2001) was 11 years of age. Gold implantation protocols in the two studies were slightly different but followed the same principle: two gold beads were implanted in five acupuncture points in Jæger et al. (2006), whilst gold wires were implanted in 3 acupuncture points in most dogs in Hielm-Björkman et al. (2001). Owner evaluation of pain was slightly different, as although visual analogue scale (VAS) was used several times to assess various pain and locomotion-related parameters in both studies, Jæger et al. (2006) included a Likert scale reflecting overall assessments of home behaviour and exercise whilst in the study by Hielm-Björkman et al. (2001), owners answered additional questionnaires regarding pain-related behaviour and locomotion. Veterinarian evaluation in both studies included a pain score and a video recorded locomotion assessment with slightly different protocols. In addition to the different sample sizes, the slightly different acupuncture protocols, as well as the evaluation methods, could have contributed to the different results.  

Neither Hielm-Björkman et al. (2001) nor Jæger et al. (2006) included an objective pain measurement in the study design. Pain in dogs cannot be directly measured due to their non-verbal nature, thus, a balanced subjective and objective measurement approach is often recommended for chronic pain measurement (Lascelles et al., 2019). Subjective pain assessments can introduce intra-observer variability (Pannucci & Wilkins, 2010), and objective pain measurements (for example, kinematic changes) in animals are indirect. However, it should be noted that objective pain measurements are not free of biases: for example, gait analysis excluding velocity data may introduce bias. Utilising expert opinion to interpret the result of multi-modal pain measurement is a reasonable way to reduce the biases from subjective and objective pain management (Lascelles et al., 2019) that can be implemented in future studies.

Gait changes could be correlated with pain or affected by other factors and ground reaction force (GRF) has been associated with inflammatory mediators (for example, prostaglandin E2) for pain (Trumble et al., 2004). Objective lameness evaluation via measurement of GRF through a gait analysis system was utilised by Teixeira et al. (2016) and Kapatkin et al. (2006), but neither study identified an improvement in lameness between treatment groups. In Kapatkin et al. (2006), such a result was in accordance with the finding of the lack of interaction between all pain-related VAS scores and the treatments.  However, in Teixeira et al. (2016), a greater improvement in the Canine Pain Brief Inventory (CBPI) (one of the three pain scores evaluated by owners) was observed in the acupuncture group (P = 0.034). The discrepancy between CBPI and GRF improvement could reflect the greater focus of CBPI on owner evaluation of pain as reflected by their dog’s behaviour, while GRF measurement focused more on lameness (Brown et al., 2013). In fact, some studies showed CBPI and GRF indices (peak vertical force [PVF], vertical impulse [VI]) could be only weakly associated or showed no association (Walton et al., 2013; and Teixeira et al., 2016), therefore, objective gait analysis is not necessarily superior to subjective pain assessment, and caregivers’ opinions should be interpreted together with the gait analysis. The gait analysis in Teixeira et al. (2016) failed to identify dogs with or without hip dysplasia, while in Kapatkin et al. (2006), the GRF analysis’s sensitivity sufficiency could not be verified due to no positive-control group being involved. It is thought that data interpretation of gait analysis is more ideal for animals with clinical signs involving a single limb (Lascelles et al., 2019), however, both studies included some dogs with pain and lameness in multiple limbs.

An overall superiority of acupuncture to placebo treatment was not identified in three out of four studies (Hielm-Björkman et al., 2001; Kapatkin et al., 2006; and Teixeira et al., 2016). In Teixeira et al. (2016), although no difference in three pain scores between the acupuncture and placebo groups  was identified, the study did find that both acupuncture and carprofen reduced lameness according to owners’ subjective evaluation, and acupuncture was associated with a decrease in the CBPI, a validated chronic pain score (CBPI: P = 0.002 for pain severity; P < 0.001 for pain interference; P < 0.001 for the total score at week 4. In Hielm-Björkman et al. (2001), the veterinarian identified a locomotion improvement in the acupuncture group (P = 0.036), as well as a decrease in signs of pain in both acupuncture and placebo groups compared with the baseline (P = 0.001 for locomotion improvement and P = 0.0034 for signs of pain). A factor that might have prevented acupuncture’s effects from being identified could be the initial variation in pain scores within each treatment group: since the pain score comparison between groups was based on the mean value, the comparison could not reflect the potential data skew within groups.

Owner pain evaluations, used in all four studies, could have been influenced by the caregiver placebo effect, through which owners are prone to believe that the placebo provided to their dog improved the dog’s chronic pain (Conzemius & Evans, 2012; and Gruen et al., 2017) during the multiple post-treatment follow-ups. Such belief could be based on multiple factors, including empathy towards their pet, optimism regarding the treatment, or access to superior health care, and may lessen the likelihood identification of a positive treatment effect of acupuncture (Lascelles et al., 2019). In addition, signs of chronic pain can wax and wane, and the pain of dogs may have been at its peak when they were enrolled in the study, with dogs in placebo groups naturally showing improvement without intervention (Lascelles et al., 2019). This manifestation of placebo effect can be mitigated by postponing the baseline data collection days or weeks later than the original inclusion screening (Lascelles et al., 2019), as can be seen in Teixeira et al. (2016).

Sham acupuncture was utilised in three of the four studies as the placebo control (Hielm-Björkman et al., 2001; Kapatkin et al., 2006; and Jæger et al., 2006). Sham acupuncture has exhibited potent placebo effects in some studies in people (Tavel, 2014), and some scholars argue it should be regarded as a positive intervention rather than an inert placebo (Briggs & Shurtleff, 2017). Considering sham acupuncture has been reported to elicit the analgesic effect of acupuncture in people (Kong et al., 2009; and Zeng et al., 2022), it might not be a valid placebo control (Wang et al., 2017) for veterinary acupuncture studies.

Individual case reports describing the success of acupuncture as monotherapy in chronic pain alleviation have emerged sporadically over the past 15 years (Chang et al., 2013; Veit, 2013; and Scognamillo-Szabó et al., 2010). The reported treatment effects in these single-case reports, however, could be attributable to the patient's individuality or other confounding elements. Finally, acupuncture is not necessarily a benign intervention. The reported adverse effects in dogs are concentrated on gold implants, and these adverse effects include soreness (Baker-Meuten et al., 2020), post-treatment bleeding and synovial leakage (Jæger et al., 2012), and inflammation (Bolliger et al., 2002), which could potentially affect a limb’s function.

Jæger et al. (2006) demonstrated efficacy of gold bead implantation for analgesia compared to a placebo, providing some evidence for the analgesic effect of acupuncture as a sole treatment. Overall, however, the very limited available current evidence suggests that although pain relief attributable to acupuncture has been recognised by owners, acupuncture alone is insufficient to alleviate chronic musculoskeletal pain and pain-related dysfunction in dogs. Being mindful of animal welfare, and acknowledging that in most clinical cases, acupuncture is utilised along with other treatments, future research regarding the analgesic effect of acupuncture in canine chronic pain should focus on assessing the efficacy of various acupuncture modalities as adjunctive analgesic therapies. Utilising a multidimensional pain evaluation approach, including carefully selected patients, applying a suitable acupuncture modality, and conducting prudent analysis will facilitate accurate measurement of various acupuncture modalities’ analgesic strength.

For dog owners who are seeking to replace medical chronic pain control with acupuncture, there is insufficient evidence of a beneficial effect for a veterinarian to recommend acupuncture as the sole pain-control therapy. Additionally, the client should be informed about the welfare concerns around not persuading further medical treatment, as well as the potential adverse effects of acupuncture therapies.

## Methodology

**Search strategy:** 

Databases searched and dates covered:	CAB Abstracts via Web of Science (1910–present). Conference Paper Index via Proquest From 1 Jan 1998 to 2 Aug 2023. Medline via Ovid (1946–present). PubMed (NCBI) 1 Jan 1998 to 2 Aug 2023. Scopus (1997–present).
Search strategy:	CAB Abstracts via Web of Science (1910–present): ((TS=(dog OR dogs OR bitch* OR canine)) AND TS=(puncture OR acupuncture* OR acupressure OR aqua-acupuncture OR aqua acupuncture OR electro acupuncture OR electro-acupuncture OR pharmacopuncture OR moxibustion OR ozone OR gold bead)) AND TS=(pain OR chronic pain OR musculoskeletal OR constant pain OR ache OR aching OR persistent pain OR arthritis OR muscle pain OR neuropathic pain OR complex regional pain OR sympathetically maintained OR phantom limb pain) Conference Paper Index via Proquest: noft(dog OR dogs OR bitch* OR canine) AND noft(puncture OR acupuncture* OR acupressure OR aqua-acupuncture OR aqua acupuncture OR electro acupuncture OR electro-acupuncture OR ozone OR pharmacopuncture OR moxibustion OR gold bead) AND noft(pain OR chronic pain OR musculoskeletal OR constant pain OR ache OR aching OR persistent pain OR arthritis OR muscle pain OR neuropathic pain OR complex regional pain OR sympathetically maintained OR phantom limb pain) Medline via Ovid (1946–present): (dog or dogs or bitch* or canine).mp. limit 1 to yr="1998 - 2023" (puncture or acupuncture* or acupressure or aqua-acupuncture or aqua acupuncture or electro acupuncture or electro-acupuncture or pharmacopuncture or ozone or moxibustion or gold bead).mp. (pain or chronic pain or musculoskeletal or constant pain or ache or aching or persistent pain or arthritis or muscle pain or neuropathic pain or complex regional pain or sympathetically maintained or phantom limb pain).mp. 1 and 2 and 3 and 4 Pubmed: (((dog[Title/Abstract] OR dogs[Title/Abstract] OR bitch*[Title/Abstract] OR canine[Title/Abstract]) AND (puncture[Title/Abstract] OR acupuncture*[Title/Abstract] OR acupressure[Title/Abstract] OR aqua-acupuncture[Title/Abstract] OR aqua acupuncture[Title/Abstract] OR electro acupuncture[Title/Abstract] OR electroacupuncture[Title/Abstract] OR pharmacopuncture[Title/Abstract] OR ozone[Title/Abstract] OR moxibustion [Title/Abstract] OR gold bead[Title/Abstract])) AND (pain[Title/Abstract] OR chronic pain[Title/Abstract] OR musculoskeletal[Title/Abstract] OR constant pain[Title/Abstract] OR ache[Title/Abstract] OR aching[Title/Abstract] OR persistent pain[Title/Abstract] OR arthritis[Title/Abstract] OR muscle pain[Title/Abstract] OR neuropathic pain[Title/Abstract] OR complex regional pain[Title/Abstract] OR sympathetically maintained[Title/Abstract] OR phantom limb pain[Title/Abstract])) AND (("1998/01/01"[Date - Publication] : "2023/08/02"[Date - Publication])) Scopus: (TITLE-ABS-KEY (dog OR dogs OR bitch* OR canine ) AND TITLE-ABS-KEY (puncture OR acupuncture* OR electro-acupuncture OR (gold AND bead) OR (aqua AND acupuncture) OR Pharmacopuncture OR moxibustion OR ozone) AND TITLE-ABS-KEY ( pain OR ( chronic AND pain ) OR musculoskeletal OR ache OR aching OR arthritis OR neuropathic OR (complex AND pain) OR ( regional AND pain ) OR ( sympathetic AND pain ) OR ( phantom AND pain))) AND PUBYEAR > 1997
Dates searches performed:	02 Aug 2023

**Exclusion / inclusion criteria:** 

Exclusion:	Non-peer-reviewed articles. Conference proceedings, books, book chapters, and letters. Articles unavailable in English. Non-clinical trials and non-placebo controlled clinical trials. Irrelevant to PICO (non-client-owned dogs, artificially induced pain, acute pain, acupuncture as adjunctive therapy). Published before 1 January 1998.
Inclusion:	Peer-reviewed journal articles, conference papers or degree thesis. Written in English. Placebo-controlled clinical trials. Relevant to PICO (client-owned dogs, pain induced by natural disease, chronic pain, acupuncture alone compared with placebo effects).

**Search outcome:** 

Database	Number of results	Excluded – Not in English	Excluded – Conference proceedings, books, book chapters, letters	Excluded – Non-clinical trial	Excluded – Irrelevant to PICO	Total relevant papers
CAB Abstracts	248	15	59	133	36	5
Proquest	100	1	0	45	50	4
Medline	103	5	2	34	58	4
PubMed	84	4	0	20	56	4
Scopus	228	26	12	122	64	4
Total relevant papers when duplicates removed						4

### ORCiD

Jianjian Gong: https://orcid.org/0000-0001-7981-0054 Mary Frances Thompson: https://orcid.org/0000-0002-5136-3982

### Conflict of interest

The authors declare no conflicts of interest.

## References

[BIBR-1] Baker-Meuten A., Wendland T., Shamir S.K., Hess A.M., Duerr F.M. (2020). Evaluation of acupuncture for the treatment of pain associated with naturally-occurring osteoarthritis in dogs: a prospective, randomized, placebo-controlled, blinded clinical trial. BMC Veterinary Research.

[BIBR-2] Bolliger C., DeCamp C.E., Stajich M., Flo G.L., Martinez S.A., Bennett R.L., Bebchuk T. (2002). Gait analysis of dogs with hip dysplasia treated with gold bead implantation acupuncture. Veterinary and Comparative Orthopaedics Traumatology.

[BIBR-3] Briggs J.P., Shurtleff D. (2017). Acupuncture and Sham Acupuncture for Pain Relief—Reply. Journal of the American Veterinary Medical Association.

[BIBR-4] Brown D.C., Boston R.C., Farrar J.T. (2013). Comparison of Force Plate Gait Analysis and Owner Assessment of Pain Using the Canine Brief Pain Inventory in Dogs with Osteoarthritis. Journal of Veterinary Internal Medicine.

[BIBR-5] Cantwell S.L. (2010). Traditional Chinese Veterinary Medicine: The Mechanism and Management of Acupuncture for Chronic Pain. Topics in Companion Animal Medicine.

[BIBR-6] Chang K.M., Chen K.S., Wang H.C., Lai C.H., Hsieh Y.T., King H.H., Lee W.M. (2013). Combination of Acupuncture and Aquapuncture Using Vitamin B Complex for Treatment of Chronic Degenerative Changes of Hip Joints and Anal Relaxation in a Dog. The Thai Journal of Veterinary Medicine.

[BIBR-7] Conzemius M.G., Evans R.B. (2012). Caregiver placebo effect for dogs with lameness from osteoarthritis. Journal of the American Veterinary Medical Association.

[BIBR-8] Gruen M.E., Dorman D.C., Lascelles B.D.X. (2017). Caregiver placebo effect in analgesic clinical trials for cats with naturally occurring degenerative joint disease-associated pain. The Veterinary Record.

[BIBR-9] Hielm-Björkman A., Raekallio M., Kuusela E., Saarto E., Markkola A., Tulamo R.M. (2001). Double-blind evaluation of implants of gold wire at acupuncture points in the dog as a treatment for osteoarthritis induced by hip dysplasia. Veterinary Record.

[BIBR-10] Jæger G.T., Larsen S., Søli N., Moe L. (2006). Double-blind, placebo-controlled trial of the pain-relieving effects of the implantation of gold beads into dogs with hip dysplasia. Veterinary Record.

[BIBR-11] Jæger G.T., Stigen Ø., Devor M., Moe L. (2012). Gold Bead Implantation in Acupoints for Coxofemoral Arthrosis in Dogs: Method Description and Adverse Effects. Animals.

[BIBR-12] Kapatkin A.S., Tomasic M., Beech J., Meadows C., Boston R.C., Mayhew P.D., Powers M.Y., Smith G.K. (2006). Effects of electrostimulated acupuncture on ground reaction forces and pain scores in dogs with chronic elbow joint arthritis. Journal of the American Veterinary Medical Association.

[BIBR-13] Kong J., Kaptchuk T.J., Polich G., Kirsch I., Vangel M., Zyloney C., Rosen B., Gollub R. (2009). Expectancy and treatment interactions: a dissociation between acupuncture analgesia and expectancy evoked placebo analgesia. NeuroImage.

[BIBR-14] Lascelles B.D.X., Brown D.C., Conzemius M.G., Gill M., Oshinsky M.L., Sharkey M. (2019). Measurement of chronic pain in companion animals: Discussions from the Pain in Animals Workshop (PAW) 2017. The Veterinary Journal.

[BIBR-15] Pannucci C.J., Wilkins E.G. (2010). Identifying and Avoiding Bias in Research. Plastic and Reconstructive Surgery.

[BIBR-16] Roynard P., Frank L., Xie H., Fowler M. (2018). Acupuncture for Small Animal Neurologic Disorders. Veterinary Clinics of North America: Small Animal Practice.

[BIBR-17] Scognamillo-Szabó M.V.R., Sousa N.R., Carvalho L.T., Carvalho F.S.R. (2010). Acupuncture and gold bead implant for hip dysplasia in German Shepherd. Acta Scientiae Veterinariae.

[BIBR-18] Tavel M.E. (2014). The Placebo Effect: the Good, the Bad, and the Ugly. The American Journal of Medicine.

[BIBR-19] Agostinho F.S., Hielm-Björkman A. (2016). Owner assessment of chronic pain intensity and results of gait analysis of dogs with hip dysplasia treated with acupuncture. Journal of the American Veterinary Medical Association.

[BIBR-20] Trumble T.N., Billinghurst R.C., McIlwraith C.W. (2004). Correlation of prostaglandin E2 concentrations in synovial fluid with ground reaction forces and clinical variables for pain or inflammation in dogs with osteoarthritis induced by transection of the cranial cruciate ligament. American Journal of Veterinary Research.

[BIBR-21] Veit N. (2013). Acupuncture as palliative pain therapy for OCD in canine shoulder joint. Zeitschrift für Ganzheitliche Tiermedizin.

[BIBR-22] Walton M.B., Cowderoy E., Lascelles D., Innes J.F. (2013). Evaluation of Construct and Criterion Validity for the ‘Liverpool Osteoarthritis in Dogs’ (LOAD) Clinical Metrology Instrument and Comparison to Two Other Instruments. PLOS ONE.

[BIBR-23] Wang Y., Zhao J., Hao D. (2017). Is sham acupuncture a real placebo: Skeptical for sham acupuncture. World Journal of Acupuncture.

[BIBR-24] Zeng D., Yan X., Deng H., Li J., Xiao J., Yuan J., Huang J., Xu N., Fu W., Liu J. (2022). Placebo response varies between different types of sham acupuncture: A randomized double-blind trial in neck pain patients. European Journal of Pain.

